# A novel α-conotoxin [D1G, ΔQ14] LvIC decreased mouse locomotor activity

**DOI:** 10.3389/fphar.2024.1466504

**Published:** 2025-01-21

**Authors:** Wen Wang, Meiting Wang, Huanbai Wang, Weifeng Xu, Conggang Wang, Jie Pei, Xiaodan Li, Dongting Zhangsun

**Affiliations:** ^1^ Key Laboratory of Tropical Biological Resources of Ministry of Education, School of Life and Health Science, Hainan University, Haikou, China; ^2^ Guangxi Key Laboratory of Special Biomedicine, School of Medicine, Guangxi University, Nanning, China

**Keywords:** α-conotoxin [D1G, ΔQ14] LvIC, locomotor activity, ncs-1, NLGN-3, nAChRs

## Abstract

**Background and Purpose:**

Nicotinic acetylcholine receptors (nAChRs), which are expressed throughout the mammalian brain, mediate a variety of physiological functions. Despite their widespread presence, the functions of nAChRs are not yet fully understood. α-Conotoxins, which are peptides derived from the venom of marine cone snails, target different subtypes of nAChRs. Specifically, α-Conotoxins [D1G, ΔQ14] LvIC, identified from *Conus lividus*, have demonstrated strong activity on α6β4* nAChRs *in vitro*. However, the effects of [D1G, ΔQ14] LvIC have not been investigated *in vivo*. This study aims to examine the activities of [D1G, ΔQ14] LvIC and explore its potential mechanisms *in vivo*.

**Methods:**

The study involved the injection of [D1G, ΔQ14] LvIC into the lateral cerebral ventricle (LV) of mice. Following this procedure, behavioral tests were conducted to assess changes in the mice’s behavior. To investigate the molecular alterations in the mice’s brains, untargeted metabolomics and label-free Liquid Chromatography-Tandem Mass Spectrometry (LC-MS/MS) were employed. Subsequently, Western blot (WB) and quantitative reverse transcription PCR (RT-qPCR) techniques were utilized to detect specific molecular changes induced by [D1G, ΔQ14] LvIC.

**Results:**

The injection of [D1G, ΔQ14] LvIC led to a decrease in locomotor activity in mice. This treatment also resulted in reduced expression of neuronal calcium sensor 1 (NCS-1) and neuroligin 3 (NLGN-3) in the prefrontal cortex (PFC), hippocampus (Hip), and caudate putamen (CPu). Both NCS-1 and NLGN-3 are crucial for neuronal development, synapse formation, and neuron activity, and their reduction is associated with decreased synapse strength. Despite these changes, results from the Morris water maze (MWM) indicated that [D1G, ΔQ14] LvIC did not impair the learning and memory abilities of the mice.

**Conclusion:**

Our findings indicate that α-conotoxin [D1G, ΔQ14] LvIC significantly decreased locomotor activity in mice. Additionally, it altered gene expression primarily in areas related to neuronal development, synapse formation, and neuron activity, while also reducing synapse strength. This study first proposed that [D1G, ΔQ14] LvIC could modulate mice’s locomotor activity. However, further investigation is needed to understand the therapeutic effects of [D1G, ΔQ14] LvIC.

## 1 Introduction

Peptide therapeutics represent a promising area in the pharmaceutical field due to their high bioavailability, potency, and reduced concerns regarding drug-drug interactions, toxicity, and tissue accumulation. Among these, α-Conotoxins have played a significant role in the pharmacological characterization of various subtypes of nicotinic acetylcholine receptors (nAChRs) both *in vivo* and *in vitro* (Nicke et al., 2004; Akondi et al., 2014). nAChRs mediated diverse physiological functions, including cognition (Dineley et al., 2015), muscle contraction (Engel et al., 2015), immunomodulation (McIntosh et al., 2009), nociception (Marvaldi et al., 2020), craving and reward (Broussard et al., 2016). However, the limited understanding of specific nAChRs subtype has hindered basic research and drug development.

In our previous study, we identified α-Conotoxin [D1G, ΔQ14] LvIC as a novel peptide derived from *Conus lividus*. [D1G, ΔQ14] LvIC could specifically block α6/α3β4 nAChRs and showed minimal or no inhibitory effect on other subtypes at 10 μM, including α1β1δε, α2β2, α2β4, α3β2, α3β4, α4β2, α4β4, α6/α3β2β3, α7, and α9α10 nAChRs *in vitro* (Zhu et al., 2023). However, it’s role is unclear *in vivo*. Our previous study showed that α-Conotoxin TxIB (block α6/α3β2β3 nAChR) could change concentration of dopamine (DA), noradrenaline (NE) and γ-aminobutyric acid (GABA) Hip and PFC of mice, and TxIB could specifically block α6/α3β2β3 nAChR (Luo et al., 2013; You et al., 2019). This finding suggests that α6 is localized in the Hip and PFC. Some reports indicated that α6/α3β4 nAChRs was associated with pain and THC dependence (Donvito et al., 2020; Knowland et al., 2020).

In addition, reports showed that NCS-1, a Ca^2+^-binding protein involved in neuroprotection, neuronal development and synapse formation (Chen et al., 2001; Zucker, 2003; Hui et al., 2007; Fischer et al., 2021). NLGN-3 could modulate neuronal activity (Venkatesh et al., 2015), synapse strength (Bemben et al., 2019), and mouse behaviors (Loos et al., 2014; Rothwell et al., 2014). In the present study, [D1G, ΔQ14] LvIC was used to investigate its potential pharmacological effects in mouse brain. In this study, [D1G, ΔQ14] LvIC was administered intracerebroventricularly to mice. Subsequent observations revealed a reduction in the mice’s locomotor activity during the open field test (OFT). Further analysis using untargeted metabolomics and proteomics of the caudate-putamen (CPu) indicated a significant decrease in the levels of NCS-1 and NLGN-3. RT-qPCR and WB results performed that the expression of NCS-1 and NLGN-3 decreased in mice’s Hip, PFC, and CPu after [D1G, ΔQ14] LvIC injection. Therefore, NCS-1 and NLGN-3 are crucial proteins for neuron functions and mouse behaviors. Thus, we speculated that the decrease of mice’s locomotor activities caused by [D1G, ΔQ14] LvIC was correlated with NCS-1 and NLGN-3 expression changes. This study first proposed that [D1G, ΔQ14] LvIC could modulate mice’s locomotor activity and had strong activity *in vivo*. However, further investigation is needed to understand the therapeutic effects of [D1G, ΔQ14] LvIC.

## 2 Materials and methods

### 2.1 Animal and peptide

**FIGURE 1:**
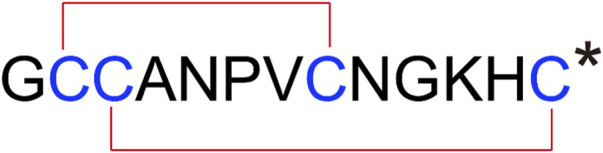
Peptide sequence of [D1G, ΔQ14] LvIC. [D1G, ΔQ14] LvIC were synthesized with Cys I−III and Cys II−IV. An asterisk (*) indicates a C-terminal amide. Glycine(G), Cysteine(C); Alanine(A); Asparagine(N); Proline(P); Valine(V); Lysine(K); Histidine(H).

Male C57BL/6J mice at 6–8 weeks of age (SJA Laboratory Animal Co., Ltd., Changsha, China) were used in this study. Mice were housed in SPF animal raising chamber of Key Laboratory of Tropical Biological Resources, Ministry of Education, University of Hainan. This study was approved by the Animal Ethics Committee of Hainan University (No.HNUAUCC-2021-00,056). Mice were randomly assigned to experimental groups, and trained experimenters were blinded to group assignments. [D1G, ΔQ14] LvIC was synthesized according to previous study. Cysteine sidechain were protected by triphenylmethyl (Trt) and acetamidomethyl (Acm). (K_3_ [Fe(CN)_6_]) and iodine oxidation were used for disulfide bond formation of [D1G, ΔQ14] LvIC (Wang et al., 2022). Sequence of [D1G, ΔQ14] LvIC was presented in Figure 1.

### 2.2 Intracerebroventricular surgery and peptide injection

A cannulae (26-gauge needle with a sleeve tubing of polyurethane) was implanted into mouse’s LV (Bregma = 0, AP: −0.6 mm, ML: +1.3 mm, DV: −2.0 mm). The detailed procedures referred to previous study (Li et al., 2021). 5 days after LV surgery, [D1G, ΔQ14] LvIC or normal saline (NS)was injected into mice’s LV.

### 2.3 Behavioral tests

**FIGURE 2:**
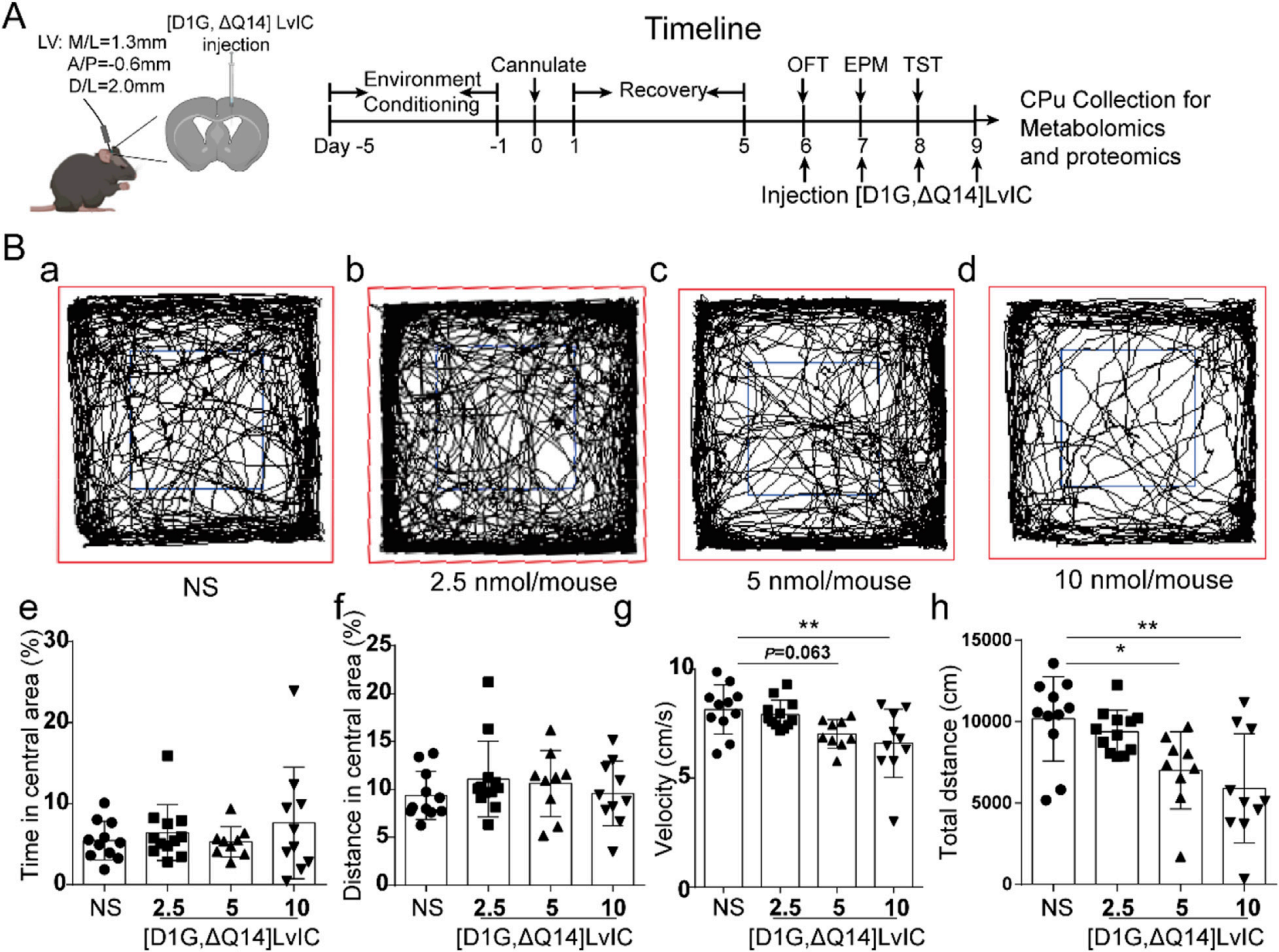
The spontaneous activity test after injection of [D1G, ΔQ14] LvIC. **(A)** Timeline of schedule. **(B)** Mice’s locomotor activity measurements in OFT test. Mice were injected into LV with the indicated drug, followed by measurement of distance traveled via video tracking software. (a-d) The trajectory of mice injected with different dose of indicated drug. Blue, center area; Red, periphery area. (e) The percentage of time in central area during 30 min test. (f) The percentage of distance in central area. (g) Velocity of the mice during the test. (h) Distance moved in the OFT test. Each group using 9–12 mice, n = 9-12. In those experiments, 42 mice were used. Data are expressed as the means ± SEM. *p < 0.05 when compared NS group, **p < 0.001 when compared NS group.

To investigate the effects of [D1G, ΔQ14] LvIC on mouse behaviors, mice were injected daily with NS or different dose of [D1G, ΔQ14] LvIC (2.5 nmol/mouse, 5 nmol/mouse, 10 nmol/mouse) into the LV for 4 days 30 min after injection, mice’s behaviors were evaluated. On fourth day, mice were sacrificed and then CPu, PFC and Hip brain regions were collected after injection. Timeline of experiments was shown in Figure 2A.

#### 2.3.1 Open field test (OFT)

After 30 min of [D1G, ΔQ14] LvIC injection, mice’s locomotor activity was recorded for 30 min (Kokkinou et al., 2021). Time in central area (%), distance in central area (%), velocity, as well as total distance were analyzed using motormonitor software (Smart 3.0, Panlab, United States).

#### 2.3.2 Elevated plus maze (EPM)

After 30 min of [D1G, ΔQ14] LvIC injection, each mouse underwent a 5 min EPM test. Time in open arms (%), distance in open arms (%) and entries in open arms were measured. Anxiety is measured as a function of decreased open arms exploration (Korte and De Boer, 2003).

#### 2.3.3 Tail suspension test (TST)

TST was performed to evaluate depression-like behaviors. After 30 min of [D1G, ΔQ14] LvIC injection, mice were suspended through tails for 6 min. Activities of mice were recorded using camera. Immobility duration total (s), Immobility duration total (%) and Immobility number total of mice were analyzed by motormonitor software (Smart 3.0, Panlab, United States).

#### 2.3.4 Rotarod test

Motor impairment was determined on the rotating rod (Ugo Basile srl, ITALY). Mice were trained before testing. Training schedule: First day, mice were placed on the rotating rod which at constant speed of 11 rpm. Second day, mice were placed on the rotating rod at constant speed of 22 rpm. At testing day (third day), after 30 min of [D1G, ΔQ14] LvIC (5 nmol/mouse) injection, mice were placed on the rotating rod of which speed rising from 4 rpm to 40 rpm in 3 min.

#### 2.3.5 Morris water maze Test (MWM)

MWM was conducted to examine mice’s ability of learning and memory, which was consisted of three different trials: visible platform testing, hidden platform testing, and probe trial (Vorhees and Williams, 2006). Water temperature was maintained about 25°C by a heating device at the bottom of pool. Mice’s activities in pool were recorded by camera and analyzed using Smart 3.0 (Panlab, United States). Experimental procedure present in Table 1.

**TABLE 1:** Experimental procedure during mouse MWM test. North (N), south (S), east (E) and west (W).

	DAY1		DAY2	DAY3	DAY4	DAY5	DAY6	DAY7
	Platform location	Starting direction	Platform location: SW Starting direction as follow					No platform
Trial 1	SW	S	N	SE	NW	E	N	NE
Trial 2	NW	N	E	NW	SE	NW	SE	
Trial 3	NE	S	SE	NW	E	N	E	
Trial 4	SE	W	NW	E	N	SE	NW	

### 2.4 Tissue collection

After 30 min of [D1G, ΔQ14] LvIC injection, mice were sacrificed and their brain tissues were collected immediately. CPu, PFC and Hip were quickly frozen in liquid nitrogen for 10 min, and then stored at −80°C for further use.

### 2.5 Untargeted metabolomics

Untargeted Metabolomics was tested by Novogene Co. Ltd. (Beijing, China). UHPLC-MS/MS analyses were performed using a Vanquish Ultra-high performance liquid chromatography (UHPLC) system (ThermoFisher, Germany) coupled with an Orbitrap Q ExactiveTMHF-X mass spectrometer (Thermo Fisher, Germany). Raw data files generated by ultra-high performance liquid chromatography coupled to mass spectrometry (UHPLC-MS/MS) were analyzed using Compound Discoverer 3.1 (CD3.1, Thermo Fisher) for each metabolite. Main parameters were set as follows: retention time tolerance, 0.2 min; actual mass tolerance, 5ppm; signal intensity tolerance, 30%; et al. After that, peak intensities were normalized to the total spectral intensity. The normalized data was used to predict the molecular formula based on additive ions, molecular ion peaks and fragment ions. And then peaks were matched with the mzCloud (https://www.mzcloud.org/).

### 2.6 Label-free LC-MS/MS

Label-free LC-MS/MS were conducted by Novogene Co. Ltd. (Beijing, China). UHPLC-MS/MS analyses were performed using a nanoElute UHPLC system (Bruker, Germany) coupled with a time TOF pro2 mass spectrometer (Bruker, Germany) in Novogene Co., Ltd. (Beijing, China).

Proteome Discoverer (Thermo, HFX and 480) or MaxQuant (Bruker, Tims) were used to tested samples. The search parameters of Proteome Discoverer were set as follows: mass tolerance for precursor ion was 10 ppm and mass tolerance for product ion was 0.02 Da. The search parameters of MaxQuant were set as follows: mass tolerance for precursor ion was 20 ppm and mass tolerance for product ion was 0.05 Da. To improve the quality of analysis results, the software PD or MaxQuant further filtered the retrieval results: Peptide Spectrum Matches (PSMs) with a credibility of more than 99% were identified PSMs.

### 2.7 Total RNA extraction and RT-qPCR

RT-qPCR was used to detect mRNA expression level of target genes. Total RNA of CPu, PFC and Hip were isolated using the TaKaRa RNAiso Reagent according to manufacturer’s instructions (TaKaRa, Dalian, China). 1 μg RNA was transcribed into cDNA using cDNA Reverse Transcription Kit (Vazyme, Nanjing, China). RT-qPCR was performed using ChamQ universal qPCR SYBR Master Mix (Vazyme, Nanjing, China). Primers sequence used in this study were listed in Table 2.

**TABLE 2:** Primers sequence used in real-time quantitative PCR.

Gene	Primer sequence (5′–3′)
*Itga-2*-F	TGT​CTG​GCG​TAT​AAT​GTT​GGC
*Itga-2*-R	CTT​GTG​GGT​TCG​TAA​GCT​GCT
*c-fos*-F	CGGGTTCAACGCCGACTA
*c-fos*-R	TTG​GCA​CTA​GAG​ACG​GAC​AG
*Gapdh*-F	AGG​TCG​GTG​TGA​ACG​GAT​TTG
*Gapdh*-R	TGT​AGA​CCA​TGT​AGT​TGA​GGT​CA
*Ncs-1*-F	GAG​GGT​GGA​CCG​GAT​CTT​TG
*Ncs-1*-R	GAG​GCT​AGT​GGT​TCC​CAC​AC
*Nlgn-3*-F	CCC​TGG​GCT​TCC​TCA​GTT​TG
*Nlgn3*-R	GGC​AAT​GGT​ACT​CTG​GCA​CC

### 2.8 Western blot assay

Mice were exposed to [D1G, ΔQ14] LvIC for 4 days, and then brain tissues were extracted protein using RIPA lysis buffer (1% PMSF) (Beyotime, China). After electrophoretic separation, proteins were transferred to polyvinylidene fluoride (PVDF) membrane. Membranes were blocked by skimmed milk powder. Then PVDF membrane incubated with primary and secondary antibodies. Protein bands were analyzed by ImageJ software (LICOR Biosciences, United States).

### 2.9 Statistical analysis

Data were analyzed using GraphPad 8.0 software, and expressed as mean ± SEM. ordinary one-way ANOVA with Tukey’s multiple comparisons test and unpaired *t*-test were used to compare the differences (*p < 0.05, **p < 0.01). SEM: Structural Equation Modeling. Two-way ANOVA was used in Figure 9b.

## 3 Results

### 3.1 Drug administration and behavioral tests

To investigate the *in vivo* functions of [D1G, ΔQ14] LvIC, it was injected into LV. 30 min after injection, mice’s locomotor activity was recorded and analyzed using OFT. Trajectory of mice was shown in Figure 2B a-d. After injecting 5 nmol/mouse [D1G, ΔQ14] LvIC, time and distance in central area (%) had no difference compared with that of control group (Figure 2B e, f). Mice velocity was not different from control values (*p* = 0.063) (Figure 2B g). Mice’s total distance was significantly reduced compared with control group (Figure 2B h).

**FIGURE 3:**
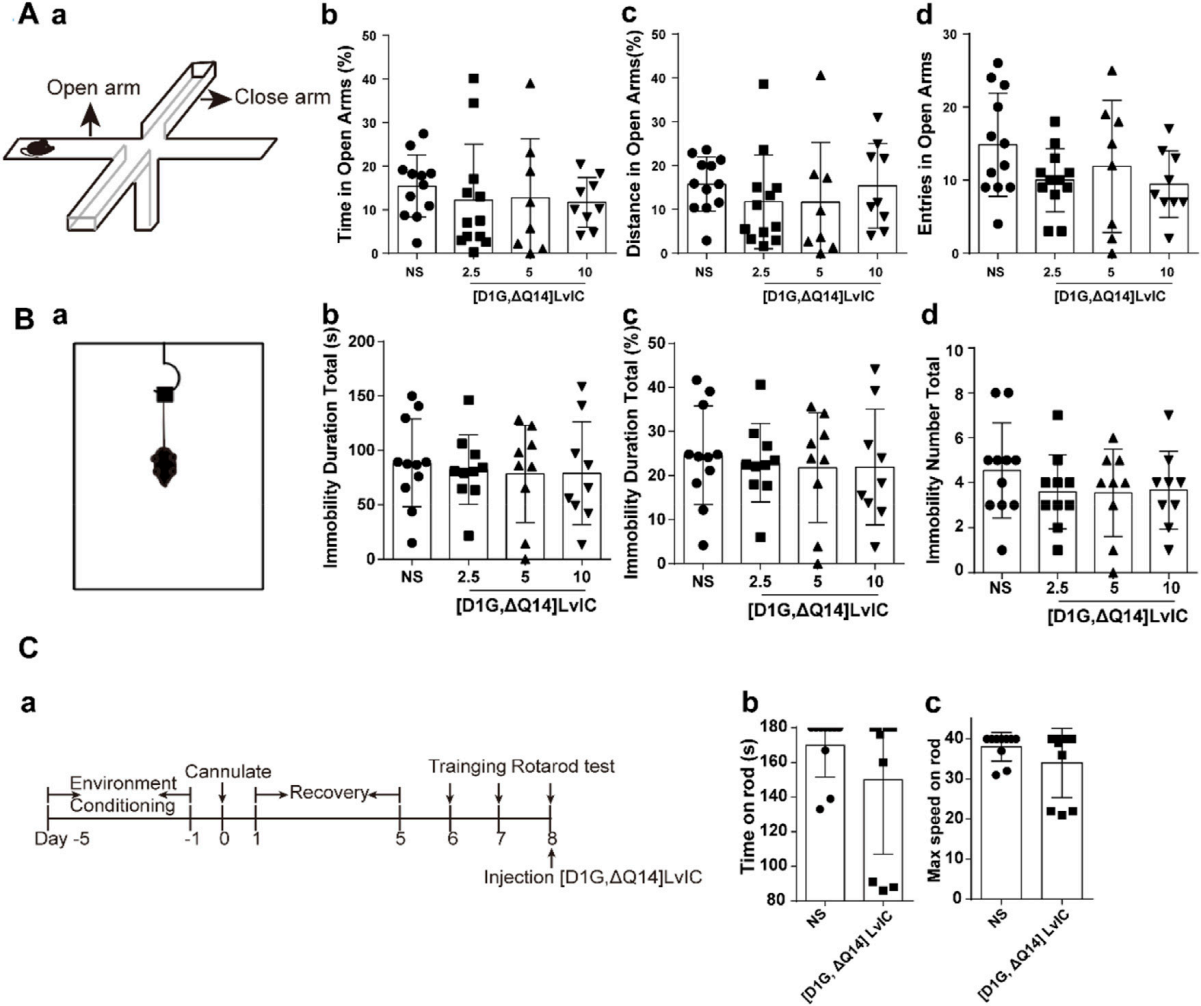
Behavioral tests after injection of [D1G, ΔQ14] LvIC. **(A)** Anxiety-like behavioral tests. (a) the diagram of EPM. (b-c) the percentage of time in open arms and the percentage of distance in open arms. (d) entries in open arms during test. **(B)** Depression-like behavioral tests. (a) the diagram of TST. (b) the immobility duration totals in TST, (c) the percentage of immobility duration totals. (d) immobility number totals during test. **(C)** Rotarod test. (a) Time-line of experiment procedures in the rotarod test (b) Time on rod. (c) the max speed when the mice drop of from the rod. Each group using 8–12 mice, n = 8-12. In those experiments, 60 mice were used. Data are expressed as the means ± SEM.

To observe changes about anxiety-like and depression-like behaviors in mice, EPM and TST were conducted. EPM results revealed no significant difference between treatment group and control group for both time in open arms (%) and distance in open arms (%) (Figure 3A b-c). Times of entries in open arms showed no significant difference between treatment group and control group (Figure 3A d). Mice’s depression-like behaviors were tested using TST. TST results showed that mice’s immobility duration total (s), immobility duration total (%) and immobility number total did not change compared with control group (Figure 3B b-d). These results indicated that [D1G, ΔQ14] LvIC did not alter mice’s anxiety-like and depression-like behaviors.

Rotarod test was used to investigate the coordination. Results showed that time on rod and the max speed on rod of mice had no significant difference between [D1G, ΔQ14] LvIC group and control group. Rotarod test results demonstrated that 5 nmol/mouse [D1G, ΔQ14] LvIC did not affect limbs coordinate of mice (Figure 3C b, c).

### 3.2 Effects of different α-conotoxins on mice

**FIGURE 4:**
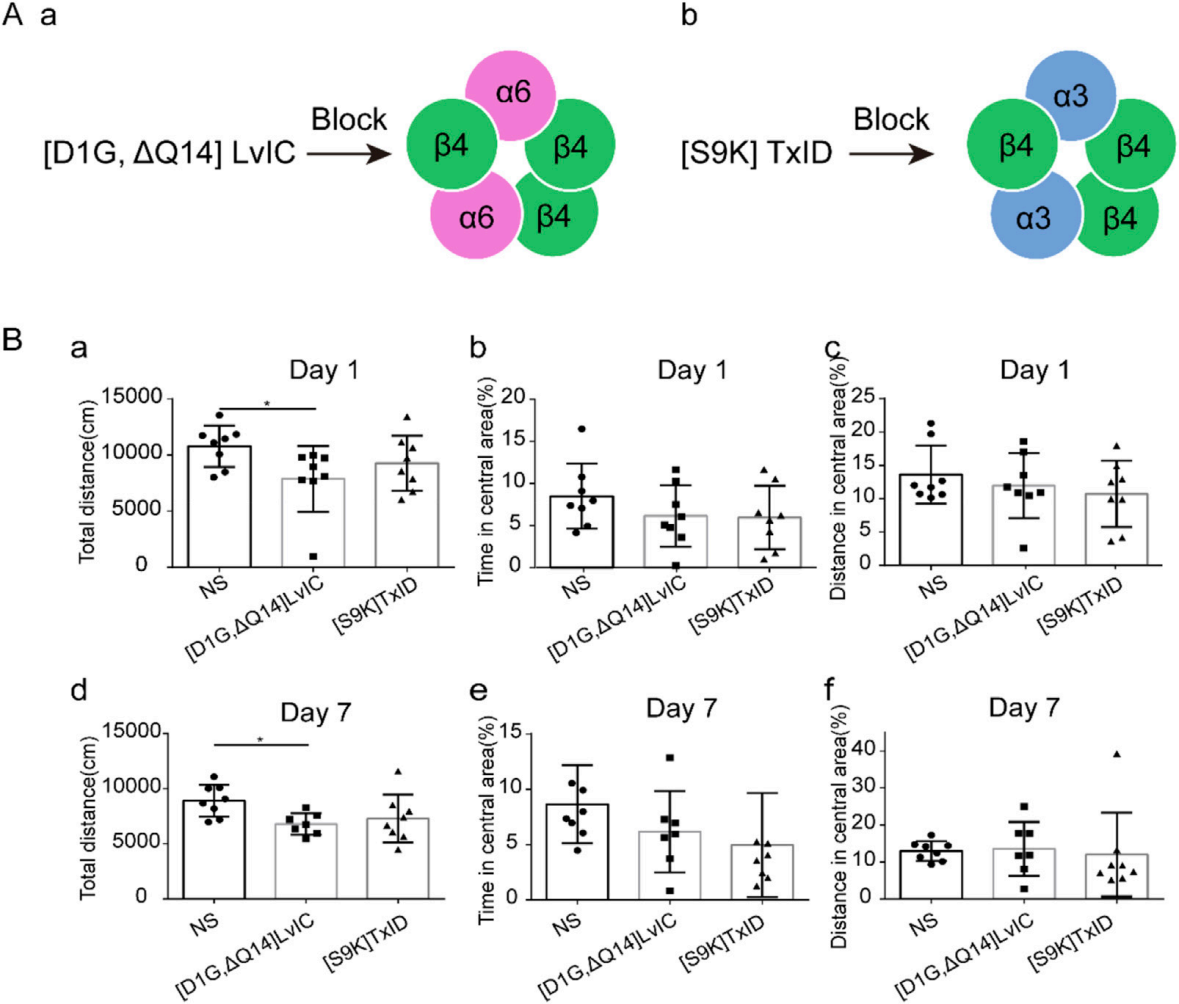
Compared different α-conotoxin targeting different nAChRs effects in OFT test. Mice were injected α-conotoxin in seven consecutive days. **(A)** α-conotoxin structure and it works on nAChRs. (a) Structure of [D1G, ΔQ14] LvIC and it works on α6β4* nAChRs. (b) Structure of [S9K] TxID and it works on α3β4* nAChRs. **(B)** the effect of [D1G, ΔQ14] LvIC and [S9K] TxID on spontaneous activity. Total distance mice traveled in the OFT test, the percentage of time in central area and the percentage of distance in central area at day 1(a-c) and day 7 (d-f). Each group using eight mice, n = 8. In those experiments, 24 mice were used. *p < 0.05 when compared NS group.

According to mice’s behavioral tests results, mice’s locomotor activity was decreased after [D1G, ΔQ14] LvIC injection. We then investigate whether the decrease of locomotor activity was specific. [S9K] TxID (Yu et al., 2018) was used to detect its’ effects on mice’s locomotor activity. Results showed [S9K] TxID could not affect mice behaviors at 5nmol/mouse (Figure 4B). These results further demonstrated that [D1G, ΔQ14] LvIC has a correlation with mice’s locomotor activity.

### 3.3 Untargeted metabolomics and label-free LC-MS/MS

**FIGURE 5:**
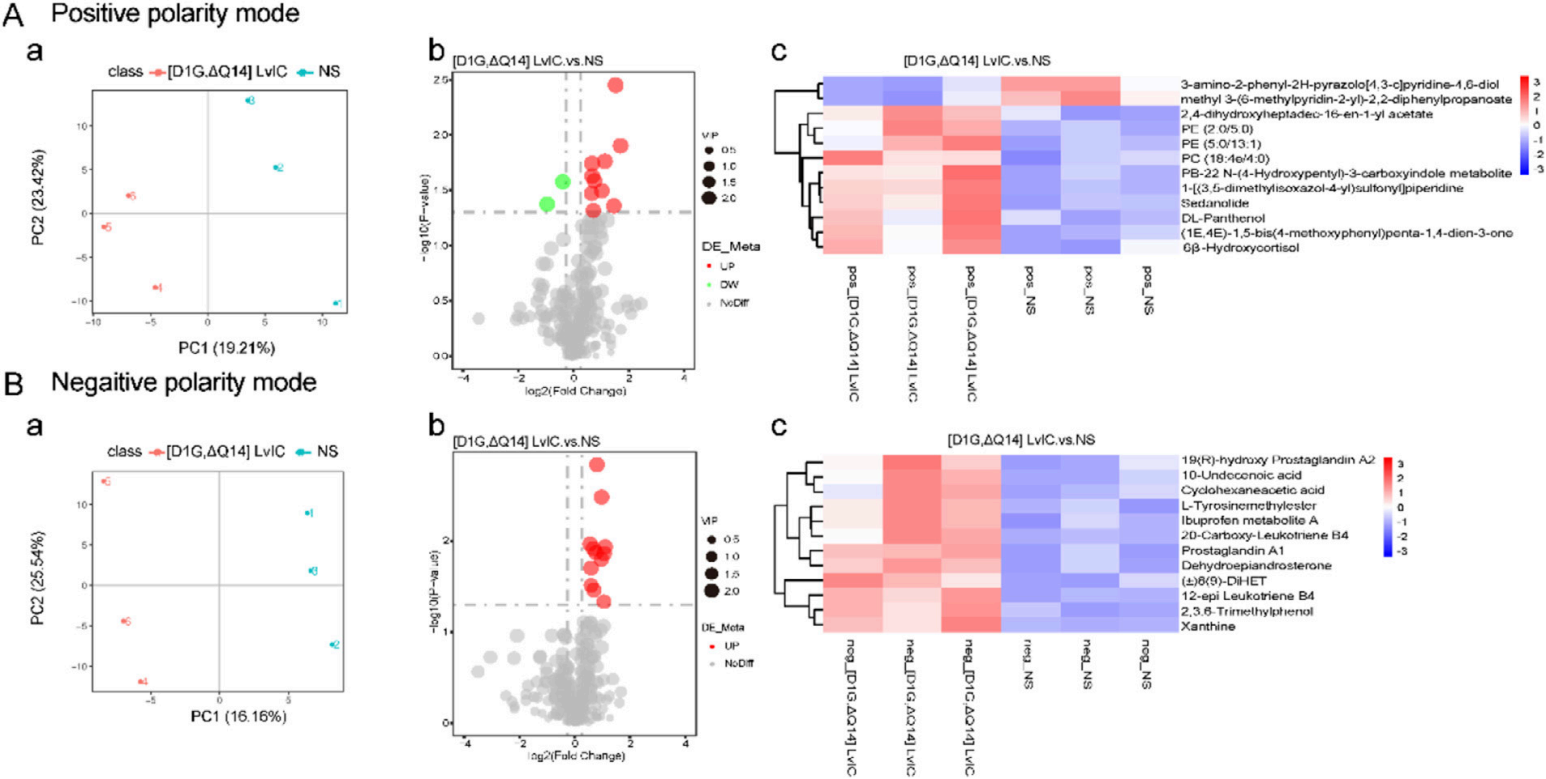
CPu untargeted metabolomics alterations induced by [D1G, ΔQ14] LvIC. **(A)** Positive polarity mode. (a) Partial least squares discriminant analysis (PLS-DA) analysis. (b) Volcanic map of differential metabolites. (c) Differential metabolite clustering heatmap. **(B)** Negative polarity mode. (a) PLS-DA analyze. (b) Volcanic map of differential metabolites. (c) Differential metabolite clustering heatmap. (Volcanic map: Green and red dot represent downregulation metabolomics and upregulation metabolomics, respectively; Heatmap: blue and red color represent downregulation metabolomics and upregulation metabolomics, respectively. Deep color means significant difference of metabolomics). Each group using nine mice, n = 9. In those experiments, 18 mice were used.

**FIGURE 6:**
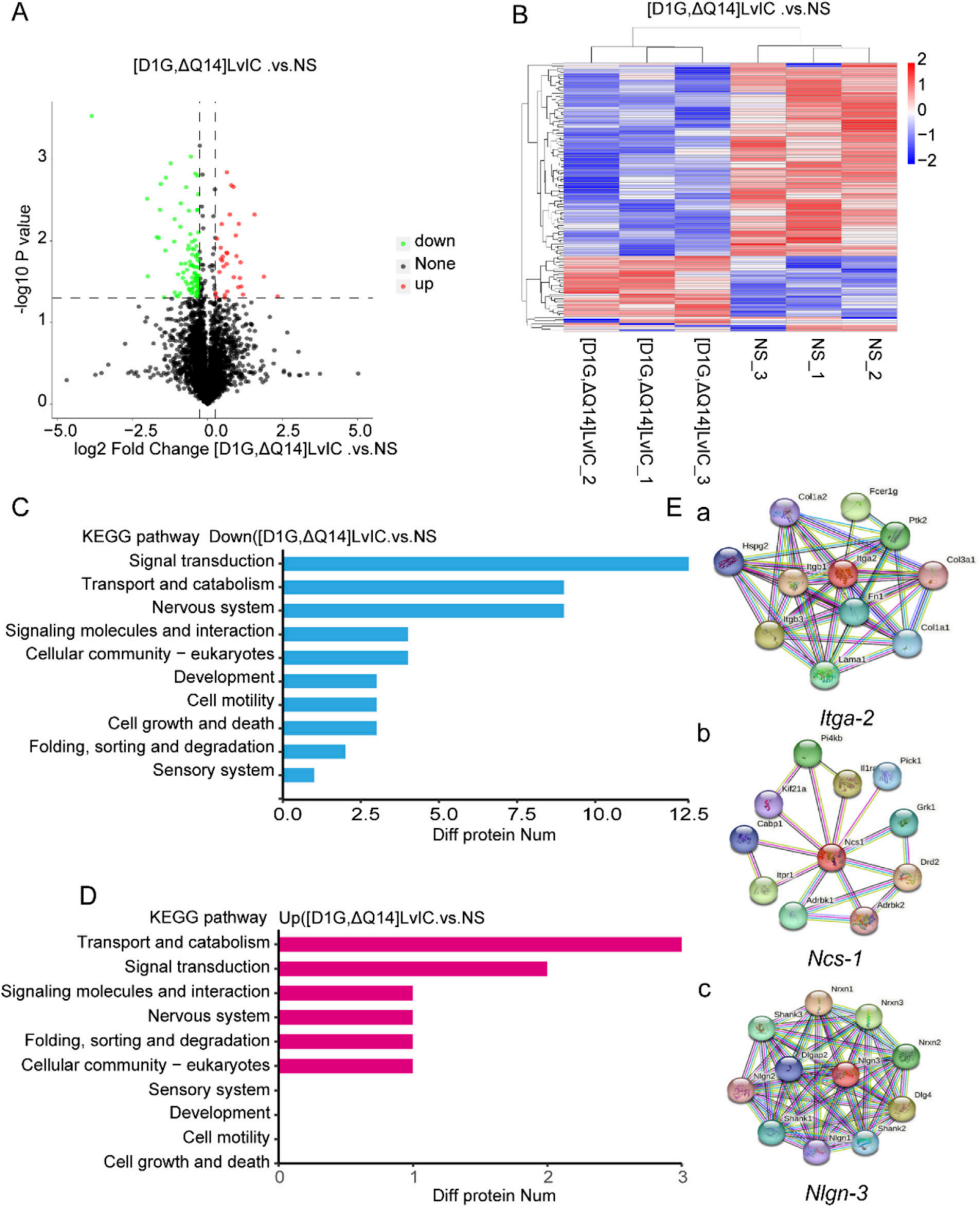
Label free LC-MS/MS analysis CPu of mice to detect the alterations of protein induced by [D1G, ΔQ14] LvIC. **(A)** Volcanic map of differential protein. **(B)** Differential protein clustering heatmap. **(C)** KEGG analysis identified the main processes of the DOWN-expressed protein. **(D)** KEGG analysis identified the main processes of the UP-expressed proteins. **(E)** Diagram of *Itga-2*, *Ncs-1*, *Nlgn-3* gene interaction network. Results from StringDB database (http://string-db.org/). (a) *Itga-2* interaction network diagram. (b) *Ncs-1* interaction network diagram. (c) *Nlgn-3* interaction network diagram. Each group using nine mice, n = 9. In those experiments, 18 mice were used.

Behavior changes in mice were usually related to molecular changes in brain. However, metabolomics results showed that there were no changes in neurotransmitters in CPu after [D1G, ΔQ14] LvIC injection (Figures 5A, B). Then label-free proteomics technology was used to screen different expression proteins. After kyoto encyclopedia of genes and genomes (KEGG) pathway analyzing, we found that the differentially expressed proteins were primarily enriched in signal transduction, transport and catabolism, and nervous system (Figures 6C, D).

Proteomics results indicated a significant decrease in NCS-1 and NLGN-3 NCS-1 and NLGN-3 expression level in mice brain after [D1G, ΔQ14] LvIC injection. These proteins interacted with several nervous system proteins (http://string-db.org/) (Figure 6E). Previous reports showed that NCS-1 correlated with growth and development of neuronal synapse (Zucker, 2003). NLGN-3 participated in the pathway of neuronal post-synaptic-signaling (https://www.cellsignal.com/pathways). The reason by which [D1G, ΔQ14] LvIC reduced NCS-1 and NLGN-3 expression remained unclear. The expression level of NCS-1 and NLGN-3 were confirmed using RT-qPCR and WB.

### 3.4 *Ncs-1, Nlgn-3, Itga-2 and c-fos* mRNA expression

**FIGURE 7:**
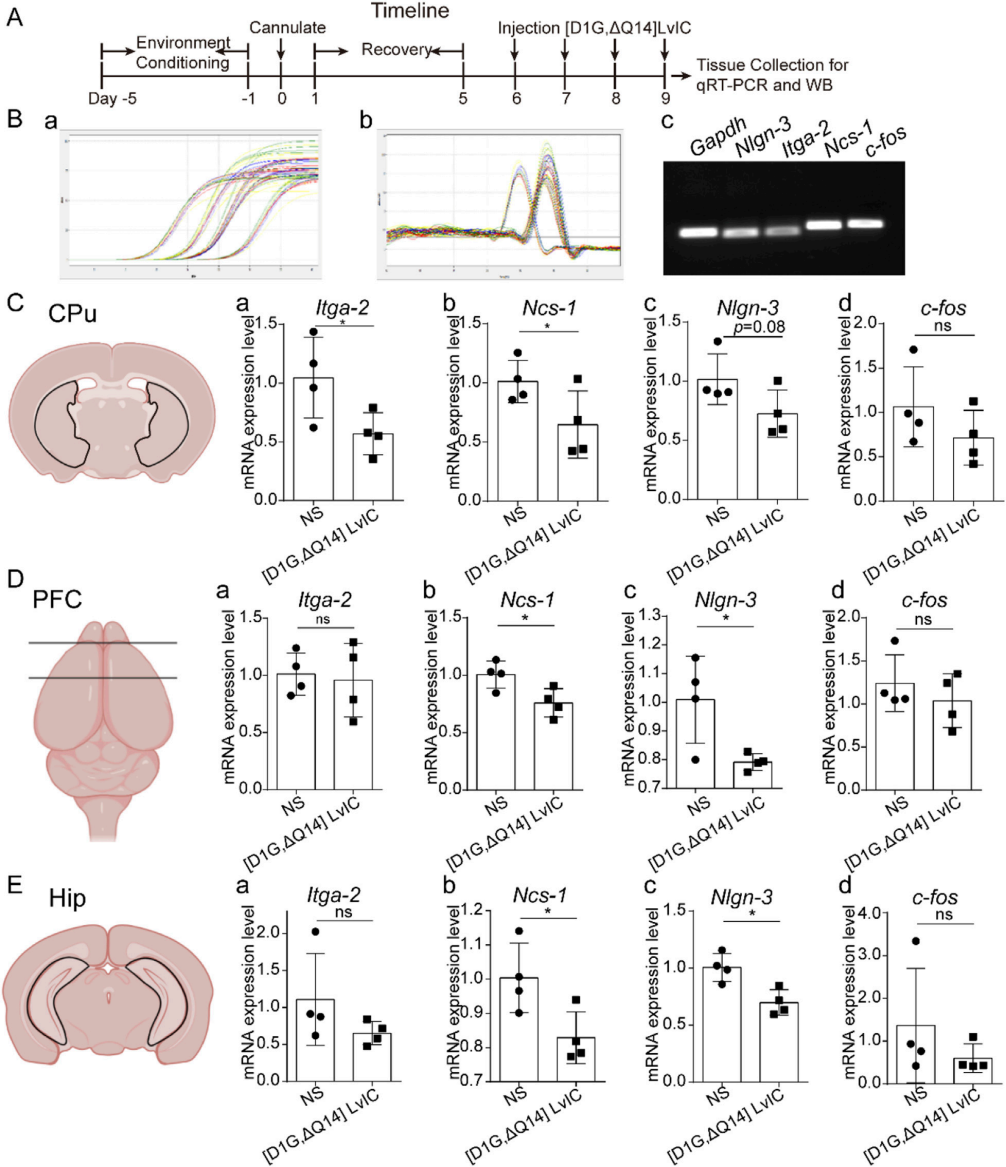
RT-qPCR detection mRNA expression. **(A)** Timeline of experiment schedule. **(B)** The specificity of primers used in the present study was verified. Amplification curve (a), melt curve (b) and electrophoretic photo (c) of qRT-PCR products. **(C)** CPu tissue mRNA expression difference detection. (a) *Itga-2*. (b) *Ncs-1*. (c) *Nlgn-3*. (d) *c-fos*. **(D)** PFC tissue mRNA expression difference detection. (a) *Itga-2*. (b) *Ncs-1*. (c) *Nlgn-3*. (d) *c-fos*. **(E)**. Hip tissue mRNA expression difference detection. (a) *Itga-2*. (b) *Ncs-1*. (c) *Nlgn-3*. (d) *c-fos*. Each group using four mice, n = 4. In those experiments, eight mice were used. Data are expressed as the means ± SEM. *p < 0.05 when compared NS group.

Total RNA was extracted from the collected tissues. Timeline was presented in Figure 7A. Primers used in these experiments were tested, and the amplification as well as melt curve were presented in Figure 7B a and Figure 7B b. The primers were highly specific to the target DNA sequences. (Figure 7B c). RT-qPCR results indicated a significant decrease in *Ncs-1* and *Itga-2* mRNA expression in CPu, while *Nlgn-3* expression showed a decrease with a *p*-value of 0.08 (Figure 7C a-c). In present study, [D1G, ΔQ14] LvIC was injected into LV of mice, potentially affecting other brain regions. Thus, mRNA expression level of *Ncs-1*, *Nlgn-3*, *Itga-2*, *c-fos* in PFC and Hip were detected. Results indicated significant reduction in *Ncs-1* and *Nlgn-3* expression in PFC and Hip (Figure 7D a-c; Figure 7E a-c). In addition, to determine whether the neuronal excitability has changed in CPu, PFC and Hip, we also detected immediately early gene *c-fos* expression which reflects neuronal excitability (Zhang et al., 2002). Results indicated that *c-fos* mRNA expression level remained unchanged (Figure 7C d, Figure 7D d, Figure 7E d).

### 3.5 NCS-1, NLGN-3, ITGA-2 and c-fos protein expression level

**FIGURE 8:**
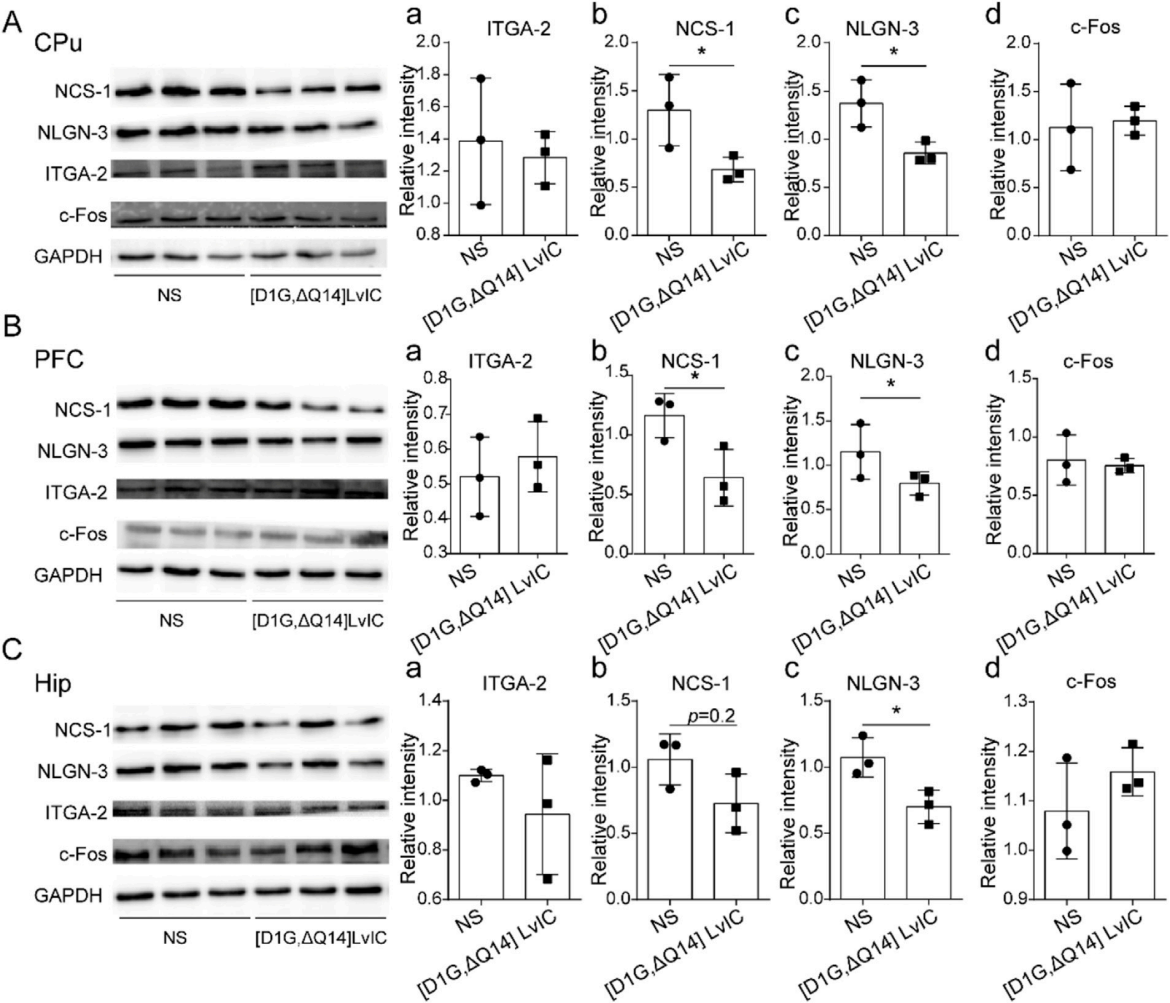
Western blot detection the gene expression. **(A)** CPu tissue protein expression difference detection. (a) ITGA-2. (b) NCS-1. (c) NLGN-3. (d) c-Fos. **(B)** PFC tissue protein expression difference detection. (a) ITGA-2. (b) NCS-1. (c) NLGN-3. (d) c-Fos. **(C)** Hip tissue protein expression difference detection. (a) ITGA-2. (b) NCS-1. (c) NLGN-3. (d) c-Fos. WB band were analyzed by ImageJ software (LICOR Biosciences, United States). Each group using three mice, n = 3. In those experiments, six mice were used. *p < 0.05 when compared NS group.

WB was used to detect the protein expression level of NCS-1, NLGN-3, ITGA-2 and c-Fos in PFC, CPu, Hip. NCS-1 and NLGN-3 exhibited a lower expression level in CPu (Figures 8A–C). Expression level of NCS-1 and NLGN-3 were reduced in PFC and Hip, respectively. (Figure 8B b-c; Figure 8C b-c). However, IGTA-2 mRNA expression decreased in CPu, while its protein expression level did not change in CPu, PFC and Hip (Figure 8A a, Figure 8B a, Figure 8C a). Besides, c-Fos protein expression level did not change in CPu, PFC and Hip (Figure 8A d, Figure 8B d, Figure 8C d). These results indicated that nerve excitability did not change after injection [D1G, ΔQ14] LvIC.

### 3.6 [D1G, ΔQ14] LvIC did not affect Mice’s learning and memory ability in MWM

**FIGURE 9:**
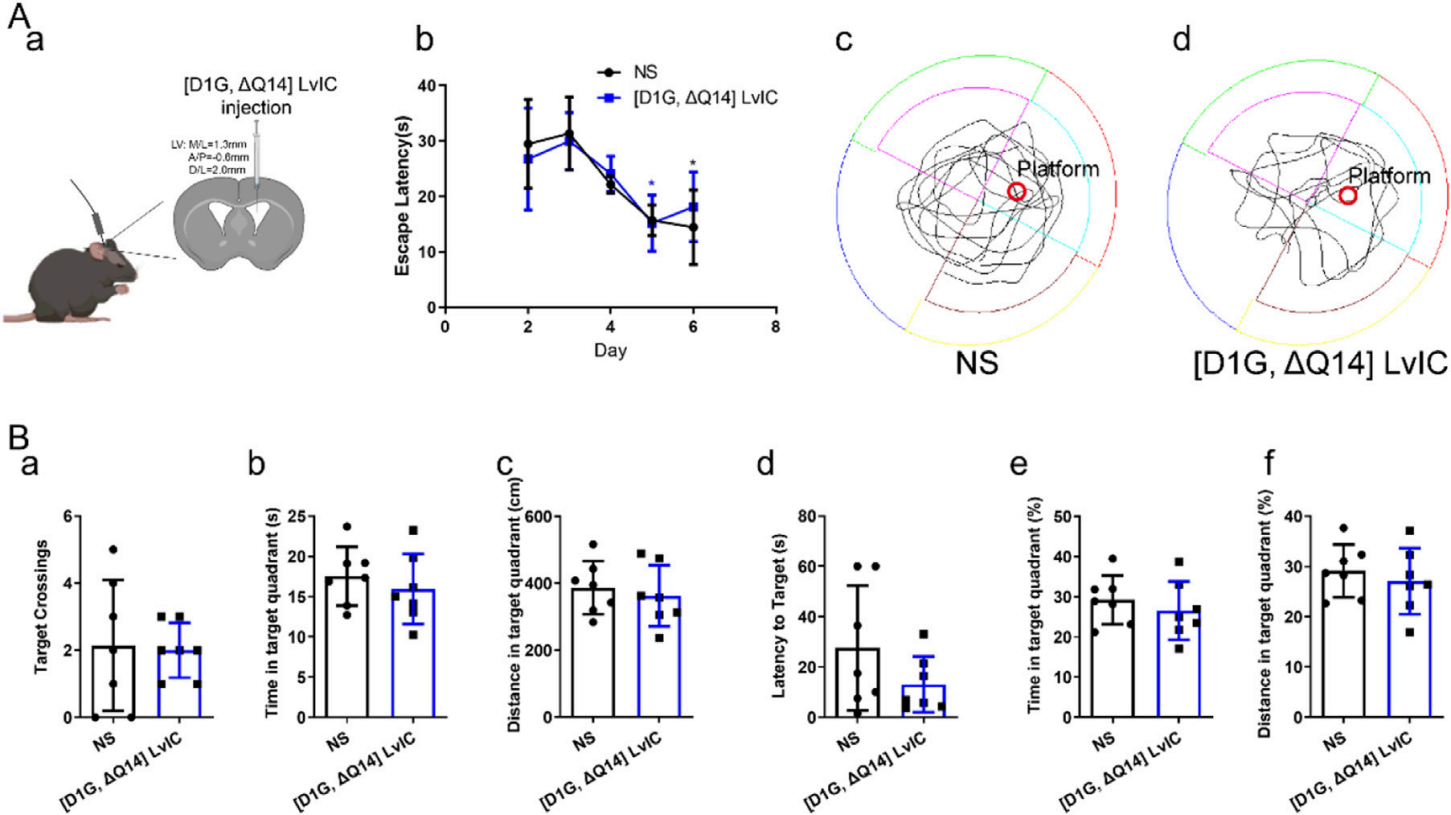
Morris Water Maze Test. **(A)** (a) Diagram of [D1G, ΔQ14] LvIC injection. (b) the escape latency during study procedure. (c) and (d) mice trajectory injected with NS and [D1G, ΔQ14] LvIC in probe trial. **(B)** During probe trial, mice’s activity was analyzed. (a) Amount of target (location of the removed platform) crossings. Time(b) and distance (c) in target quadrant. (d) latency of first time to target. and percentage of time (e) and distance (f) in target quadrant. Each group using seven mice, n = 7. In those experiments, 14 mice were used. Blue *p < 0.05 when compared second day in [D1G, ΔQ14] LvIC group, Black *p < 0.05 when compared second day in NS group.

Proteins of NCS-1 and NLGN-3 in mice’s Hip were reduced after injection of [D1G, ΔQ14] LvIC into mice’s LV. Reported studies demonstrated that NCS-1 had effects on neuronal synapses in Hip (Sippy et al., 2003). And Hip was a crucial brain region correlated with mice’s learning and memory. Therefore, we tested mice’s learning and memory ability using MWM experiments over a period of 7 days. In MWM testing, [D1G, ΔQ14] LvIC was injected 30 min before the test each day. Results indicated no statistically significant difference in escape latency difference compared with control groups during hiding platform trial (Figure 9A b). During probe trial, mice’s trajectory was analyzed (Figure 9A c, d). Results demonstrated that target crossings (Figure 9B, a), time and distance in target quadrant (Figure 9B, b, c), latency of to target (Figure 9B d), percentage of time and distance in target quadrant (Figure 9B e, f) of mice were similar between mice treated with [D1G, ΔQ14] LvIC or those treated with NS. These results indicated that continuous injection of [D1G, ΔQ14] LvIC did not affected mice’s learning and memory ability.

## 4 Discussion

α-Conotoxins are the largest group of venom peptides isolated from cone snail venoms. They could block nAChRs, but their function has not been thoroughly investigated *in vivo*. [D1G, ΔQ14] LvIC is a novel α-conotoxin peptide. In present study, we investigated pharmacological effects of [D1G, ΔQ14] LvIC *in vivo.* After [D1G, ΔQ14] LvIC LV injection, results showed that mice’s depression-like, anxiety-like, limb cooperation behaviors were not affected. However, their locomotor activity was reduced (Figure 1). To investigate whether mice’s locomotor activity changes were specific, [S9K] TxID was used to detect its effects on mice’s locomotor activity. [S9K] TxID did not change mice’s total distance in OFT. These illustrated that α6β4* nAChRs play an important role in locomotor activity changes.

**FIGURE 10:**
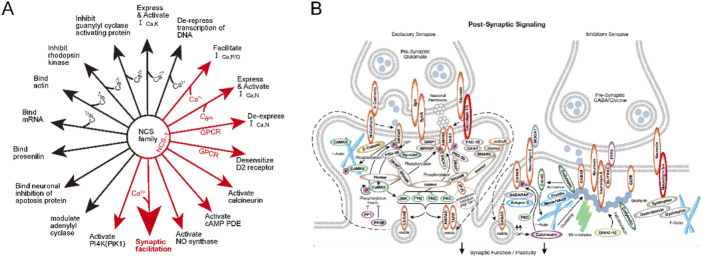
The Functions of NCS-1 and the NLGN-3 function in pathway. **(A)** The function of NCS family. the Big red arrow show that NCS-1 correlated with synaptic facilitation. **(B)**. post-synaptic signaling pathway. The NLGN-3 were marked with red circle. More detail explanation could see on the website (https://www.cellsignal.com/pathways).

Label-free LC-MS/MS results showed that NCS-1 and NLGN-3 were significantly reduced after [D1G, ΔQ14] LvIC injection. Reports proved that Ca_v_1.3 L-type-Ca^2+^ channels and D2-autoreceptor, controlled by NCS-1, contribute to Parkinson’s disease (Borgkvist et al., 2014; Dragicevic et al., 2014). Ca_v_2.3 deficiency upregulated transcripts for NCS-1. Conversely, NCS-1 knockout exacerbated neurodegeneration and downregulated Ca_v_2.3 (Benkert et al., 2019). Therefore, NCS-1 may be a potential target for motor disorder treatment. Moreover, Sippy et al. presented that NCS-1 mediated synaptic facilitation at excitatory synapses in rat hippocampal cell (Sippy et al., 2003). Fischer and Kwokyin et al. proved that NCS-1 modulated gene expression that related to neuronal morphology and development (Hui et al., 2007; Fischer et al., 2021). Furthermore, Overexpression NCS-1 in rodent NG108-15 cells enhances synapse formation and transmission (Chen et al., 2001). Some functions of NCS-1 were sorted in Figure 10A (Zucker, 2003).

Neuroligins (NLGN) are a family of postsynaptic cell-adhesion molecules, playing vital roles in synaptogenesis through their neurexins ligands. In addition, NLGN are known to drive postsynaptic assembly through binding to PSD-95 (Shipman et al., 2011). Interestingly, NLGN-3 expresses at inhibitory and excitatory synapses, enabling it to modulate both inhibitory and excitatory synaptic transmission. R451C KI mice caused NLGN-3 expression decreased could increase inhibitory synaptic strength and exhibited impaired social behaviors (Budreck and Scheiffele, 2007; Tabuchi et al., 2007). Besides, NLGN-3 degradation could reduce synapse strength in neurons (Bemben et al., 2019). Thus, NLGN-3 show strong relationship with neuronal functions (Venkatesh et al., 2015; Venkatesh et al., 2017). Some functions of NLGN-3 were sorted in Figure 10B (https://www.cellsignal.com/pathways). NCS-1and NLGN-3 affect functions of synapse which maybe the reason for mice’s behavior changes.

In conclusion, Results of this study showed that [D1G, ΔQ14] LvIC could reduce mice’s locomotor activity and NCS-1, NLGN-3 expression in mouse brain. Thus, [D1G, ΔQ14] LvIC is a potential new peptide for modulating neuron development and synapse strength. Finally, this study was unable to elucidate how the [D1G, ΔQ14] LvIC leaded to a decrease in the expression of the NCS-1 and NLGN-3 protein. Additionally, it remains unclear which functions of NCS-1 and NLGN-3 changes were responsible for the observed behavioral changes in mice. Therefore, further research is necessary to explore the mechanisms and implications of the [D1G, ΔQ14] LvIC.

This study initially investigated the effects and mechanisms of [D1G, ΔQ14] LvIC *in vivo*. The results showed that [D1G, ΔQ14] LvIC could decrease mouse locomotor activity specifically compared with [S9K] TXID. In addition, we examined c-Fos protein in PFC, CPu and Hip region. Results indicated that neuronal activity was not affected by [D1G, ΔQ14] LvIC. Furthermore, our findings demonstrated a decrease in the levels of NCS-1 and NLGN-3 in the CPu, PFC, and Hip, which could account for the observed reduction in locomotor activity in mice. Ultimately, this study concluded that [D1G, ΔQ14] LvIC did not impair the learning and memory abilities of the mice.

## Data Availability

The mass spectrometry proteomics data have been deposited to the ProteomeXchange Consortium, via the iProX partner repository with the dataset identifier PXD059357. Available at https://proteomecentral.proteomexchange.org/cgi/GetDataset?ID=PXD059357.

## Abbreviations

nAChRs, Nicotinic acetylcholine receptors; LV, Lateral cerebral ventricle; WB, Western blot; RT-qPCR, quantitative reverse transcription PCR; NCS-1, Neuronal calcium sensor 1; NLGN-3, Neuroligin 3; IGTA-2, Integrin alpha-2; PFC, Prefrontal cortex; Hip, Hippocampus; CPu, Caudate putamen; OFT, Open filed test; Trt, triphenylmethyl; Acm, Acetamidomethyl; NS, Normal saline; OFT, Open field test; TST, Tail suspension test; EPM, Elevated plus maze; MWM, Morris water maze Test; UHPLC, Ultra-high performance liquid chromatography; UHPLC-MS/MS, Ultra-high performance liquid chromatography coupled to mass spectrometry; N, North; S, South; E, East; W, West; KEGG, Kyoto encyclopedia of genes and genomes; PLS-DA, Partial least squares discriminant analysis; PSMs, Peptide Spectrum Matches.

## References

[B1] Akondi K. B. Muttenthaler M. Dutertre S. Kaas Q. Craik D. J. Lewis R. J. (2014). Discovery, synthesis, and structure-activity relationships of conotoxins. Chem. Rev. 114, 5815–5847. 10.1021/cr400401e 24720541 PMC7610532

[B2] Bemben M. A. Nguyen T. A. Li Y. Wang T. Nicoll R. A. Roche K. W. (2019). Isoform-specific cleavage of neuroligin-3 reduces synapse strength. Mol. Psychiatry 24, 145–160. 10.1038/s41380-018-0242-y 30242227

[B3] Benkert J. Hess S. Roy S. Beccano-Kelly D. Wiederspohn N. Duda J. (2019). Cav2.3 channels contribute to dopaminergic neuron loss in a model of Parkinson's disease. Nat. Commun. 10, 5094. 10.1038/s41467-019-12834-x 31704946 PMC6841684

[B4] Borgkvist A. Mosharov E. V. Sulzer D. (2014). Calcium currents regulate dopamine autoreceptors. Brain 137, 2113–2115. 10.1093/brain/awu150 25057130 PMC4571140

[B5] Broussard J. I. Yang K. Levine A. T. Tsetsenis T. Jenson D. Cao F. (2016). Dopamine regulates aversive contextual learning and associated *in vivo* synaptic plasticity in the Hippocampus. Cell Rep. 14, 1930–1939. 10.1016/j.celrep.2016.01.070 26904943 PMC4772154

[B6] Budreck E. C. Scheiffele P. (2007). Neuroligin-3 is a neuronal adhesion protein at GABAergic and glutamatergic synapses. Eur. J. Neurosci. 26, 1738–1748. 10.1111/j.1460-9568.2007.05842.x 17897391

[B7] Chen X. L. Zhong Z. G. Yokoyama S. Bark C. Meister B. Berggren P. O. (2001). Overexpression of rat neuronal calcium sensor-1 in rodent NG108-15 cells enhances synapse formation and transmission. J. Physiol. 532, 649–659. 10.1111/j.1469-7793.2001.0649e.x 11313436 PMC2278582

[B8] Dineley K. T. Pandya A. A. Yakel J. L. (2015). Nicotinic ACh receptors as therapeutic targets in CNS disorders. Trends Pharmacol. Sci. 36, 96–108. 10.1016/j.tips.2014.12.002 25639674 PMC4324614

[B9] Donvito G. Muldoon P. P. Jackson K. J. Ahmad U. Zaveri N. T. Mcintosh J. M. (2020). Neuronal nicotinic acetylcholine receptors mediate (9) -THC dependence: mouse and human studies. Addict. Biol. 25, e12691. 10.1111/adb.12691 30378732 PMC6509006

[B10] Dragicevic E. Poetschke C. Duda J. Schlaudraff F. Lammel S. Schiemann J. (2014). Cav1.3 channels control D2-autoreceptor responses via NCS-1 in substantia nigra dopamine neurons. Brain 137, 2287–2302. 10.1093/brain/awu131 24934288 PMC4107734

[B11] Engel A. G. Shen X.-M. Selcen D. Sine S. M. (2015). Congenital myasthenic syndromes: pathogenesis, diagnosis, and treatment. Lancet Neurology 14, 461–434. 10.1016/S1474-4422(15)00010-1 25895926

[B12] Fischer T. T. Nguyen L. D. Ehrlich B. E. (2021). Neuronal calcium sensor 1 (NCS1) dependent modulation of neuronal morphology and development. Faseb J. 35, e21873. 10.1096/fj.202100731R 34499766 PMC8462996

[B13] Hui K. Fei G. H. Saab B. J. Su J. Roder J. C. Feng Z. P. (2007). Neuronal calcium sensor-1 modulation of optimal calcium level for neurite outgrowth. Development 134, 4479–4489. 10.1242/dev.008979 18039973

[B14] Knowland D. Gu S. Eckert W. A. Dawe G. B. Matta J. A. Limberis J. (2020). Functional α6β4 acetylcholine receptor expression enables pharmacological testing of nicotinic agonists with analgesic properties. J. Clin. Invest 130, 6158–6170. 10.1172/JCI140311 33074244 PMC7598046

[B15] Kokkinou M. Irvine E. E. Bonsall D. R. Natesan S. Wells L. A. Smith M. (2021). Reproducing the dopamine pathophysiology of schizophrenia and approaches to ameliorate it: a translational imaging study with ketamine. Mol. Psychiatry 26, 2562–2576. 10.1038/s41380-020-0740-6 32382134 PMC8440182

[B16] Korte S. M. De Boer S. F. (2003). A robust animal model of state anxiety: fear-potentiated behaviour in the elevated plus-maze. Eur. J. Pharmacol. 463, 163–175. 10.1016/s0014-2999(03)01279-2 12600708

[B17] Li X. Xiong J. Zhang B. Zhangsun D. Luo S. (2021). α-Conotoxin TxIB inhibits development of morphine-induced conditioned place preference in mice *via* blocking α6β2* nicotinic acetylcholine receptors. Front. Pharmacol. 12, 772990. 10.3389/fphar.2021.772990 34925031 PMC8681874

[B18] Loos M. Mueller T. Gouwenberg Y. Wijnands R. Van Der Loo R. J. Birchmeier C. (2014). Neuregulin-3 in the mouse medial prefrontal cortex regulates impulsive action. Biol. Psychiatry 76, 648–655. 10.1016/j.biopsych.2014.02.011 24703509

[B19] Luo S. Zhangsun D. Wu Y. Zhu X. Hu Y. Mcintyre M. (2013). Characterization of a novel α-conotoxin from conus textile that selectively targets α6/α3β2β3 nicotinic acetylcholine receptors. J. Biol. Chem. 288, 894–902. 10.1074/jbc.M112.427898 23184959 PMC3543038

[B20] Marvaldi L. Panayotis N. Alber S. Dagan S. Y. Okladnikov N. Koppel I. (2020). Importin α3 regulates chronic pain pathways in peripheral sensory neurons. Science 369 (6505), 842–846. 10.1126/science.aaz5875 32792398

[B21] Mcintosh J. M. Absalom N. Chebib M. Elgoyhen A. B. Vincler M. (2009). Alpha9 nicotinic acetylcholine receptors and the treatment of pain. Biochem. Pharmacol. 78, 693–702. 10.1016/j.bcp.2009.05.020 19477168 PMC2739401

[B22] Nicke A. Wonnacott S. Lewis R. J. (2004). Alpha-conotoxins as tools for the elucidation of structure and function of neuronal nicotinic acetylcholine receptor subtypes. Eur. J. Biochem. 271, 2305–2319. 10.1111/j.1432-1033.2004.04145.x 15182346

[B23] Rothwell P. E. Fuccillo M. V. Maxeiner S. Hayton S. J. Gokce O. Lim B. K. (2014). Autism-associated neuroligin-3 mutations commonly impair striatal circuits to boost repetitive behaviors. Cell 158, 198–212. 10.1016/j.cell.2014.04.045 24995986 PMC4120877

[B24] Shipman S. L. Schnell E. Hirai T. Chen B. S. Roche K. W. Nicoll R. A. (2011). Functional dependence of neuroligin on a new non-PDZ intracellular domain. Nat. Neurosci. 14, 718–726. 10.1038/nn.2825 21532576 PMC3171182

[B25] Sippy T. Cruz-Martín A. Jeromin A. Schweizer F. E. (2003). Acute changes in short-term plasticity at synapses with elevated levels of neuronal calcium sensor-1. Nat. Neurosci. 6, 1031–1038. 10.1038/nn1117 12947410 PMC3132582

[B26] Tabuchi K. Blundell J. Etherton M. R. Hammer R. E. Liu X. Powell C. M. (2007). A neuroligin-3 mutation implicated in autism increases inhibitory synaptic transmission in mice. Science 318, 71–76. 10.1126/science.1146221 17823315 PMC3235367

[B27] Venkatesh H. S. Johung T. B. Caretti V. Noll A. Tang Y. Nagaraja S. (2015). Neuronal activity promotes glioma growth through neuroligin-3 secretion. Cell 161, 803–816. 10.1016/j.cell.2015.04.012 25913192 PMC4447122

[B28] Venkatesh H. S. Tam L. T. Woo P. J. Lennon J. Nagaraja S. Gillespie S. M. (2017). Targeting neuronal activity-regulated neuroligin-3 dependency in high-grade glioma. Nature 549, 533–537. 10.1038/nature24014 28959975 PMC5891832

[B29] Vorhees C. V. Williams M. T. (2006). Morris water maze: procedures for assessing spatial and related forms of learning and memory. Nat. Protoc. 1, 848–858. 10.1038/nprot.2006.116 17406317 PMC2895266

[B30] Wang L. Wu X. Zhu X. Zhangsun D. Wu Y. Luo S. (2022). A novel α4/7-conotoxin QuIA selectively inhibits α3β2 and α6/α3β4 nicotinic acetylcholine receptor subtypes with high efficacy. Mar. Drugs 20, 146. 10.3390/md20020146 35200675 PMC8878501

[B31] You S. Li X. Xiong J. Zhu X. Zhangsun D. Zhu X. (2019). α-Conotoxin TxIB: a uniquely selective ligand for α6/α3β2β3 nicotinic acetylcholine receptor attenuates nicotine-induced conditioned place preference in mice. Mar. Drugs 17, 490. 10.3390/md17090490 31443523 PMC6780885

[B32] Yu J. Zhu X. Harvey P. J. Kaas Q. Zhangsun D. Craik D. J. (2018). Single amino acid substitution in α-conotoxin TxID reveals a specific α3β4 nicotinic acetylcholine receptor antagonist. J. Med. Chem. 61, 9256–9265. 10.1021/acs.jmedchem.8b00967 30252466

[B33] Zhang J. Zhang D. Mcquade J. S. Behbehani M. Tsien J. Z. Xu M. (2002). c-fos regulates neuronal excitability and survival. Nat. Genet. 30, 416–420. 10.1038/ng859 11925568

[B34] Zhu X. Wang S. Kaas Q. Yu J. Wu Y. Harvey P. J. (2023). Discovery, characterization, and engineering of LvIC, an α4/4-conotoxin that selectively blocks rat α6/α3β4 nicotinic acetylcholine receptors. J. Med. Chem. 66, 2020–2031. 10.1021/acs.jmedchem.2c01786 36682014

[B35] Zucker R. S. (2003). NCS-1 stirs somnolent synapses. Nat. Neurosci. 6, 1006–1008. 10.1038/nn1003-1006 14513033

